# Solitary Fibrous Tumor of the Sigmoid Colon Masquerading as an Adnexal Neoplasm

**DOI:** 10.1155/2016/4182026

**Published:** 2016-09-08

**Authors:** Laura Bratton, Rabih Salloum, Wenqing Cao, Aaron R. Huber

**Affiliations:** ^1^Department of Surgical Pathology, University of Rochester Medical Center, 601 Elmwood Avenue, Box 626, Rochester, NY 14642, USA; ^2^Department of Colorectal Surgery, University of Rochester Medical Center, 601 Elmwood Avenue, Rochester, NY 14642, USA; ^3^Department of Pathology, NYU Langone Medical Center, 550 1st Ave, Suite TH388, New York, NY 10016, USA

## Abstract

Solitary fibrous tumor is a rare, benign spindle cell neoplasm that was first described in the thoracic pleura. This tumor is now known to occur at many extrapleural sites. There are established criteria for the diagnosis of malignant solitary fibrous tumor including ≥4 mitotic figures per 10 high-power fields, increased cellularity, cytologic atypia, infiltrative margins, and/or necrosis. Although all solitary fibrous tumors have the potential to recur or metastasize, those with malignant histologic features tend to behave more aggressively. We report a case of solitary fibrous tumor, with malignant histologic features, in a 21-year-old woman which arose from the serosal surface of the sigmoid colon.

## 1. Background

Solitary fibrous tumor is a rare spindle cell neoplasm that most commonly arises from the thoracic pleura; however, solitary fibrous tumor is increasingly being reported to arise in multiple extrapleural anatomic sites including the soft tissues of the head and neck, thoracic wall, mediastinum, pericardium, retroperitoneum, peritoneum, abdomen, meninges, orbit, upper respiratory tract, salivary glands, thyroid, liver, adrenal gland, kidney, spermatic cord, urinary bladder, prostate, uterine cervix, spinal cord, and periosteum [1–9]. Most extrapleural solitary fibrous tumors occur in adults between 20 and 70 years of age and tend to occur equally in men and women [1–9]. These are traditionally benign, slow growing tumors that often remain asymptomatic until they compress other structures producing pain, urinary obstruction or retention, bowel obstruction or constipation, a palpable mass, and neurologic or vascular symptoms [1, 5]. Rarely, a paraneoplastic syndrome of hypoglycemia occurs due to tumor production of insulin growth factor 2 (IGF2), which is more common in the tumors of the retroperitoneum and liver [1, 5].

These rare mesenchymal derived spindle neoplasms are currently classified as “typical” or “malignant.” The criteria to designate the tumor as malignant are dependent on the number of mitoses (≥4/10 high-power fields), cellular atypia, presence of necrosis, hypercellularity, and/or infiltrative margins [1–10]. Additionally, there is usually the presence of a hemangiopericytoma-like vascular pattern and positivity for CD34 and Bcl-2 by immunohistochemistry [1–10]. Surgical excision is the treatment of choice with a 5-year survival close to 100% if completely resected. However, malignant histology is the best predictor of poor outcome [1–3, 5–10]. We describe a case of an intra-abdominal malignant solitary fibrous tumor apparently arising from the serosal surface of the sigmoid colon presenting as an adnexal mass.

## 2. Case Presentation

### 2.1. Clinical Summary

**Figure 1:**
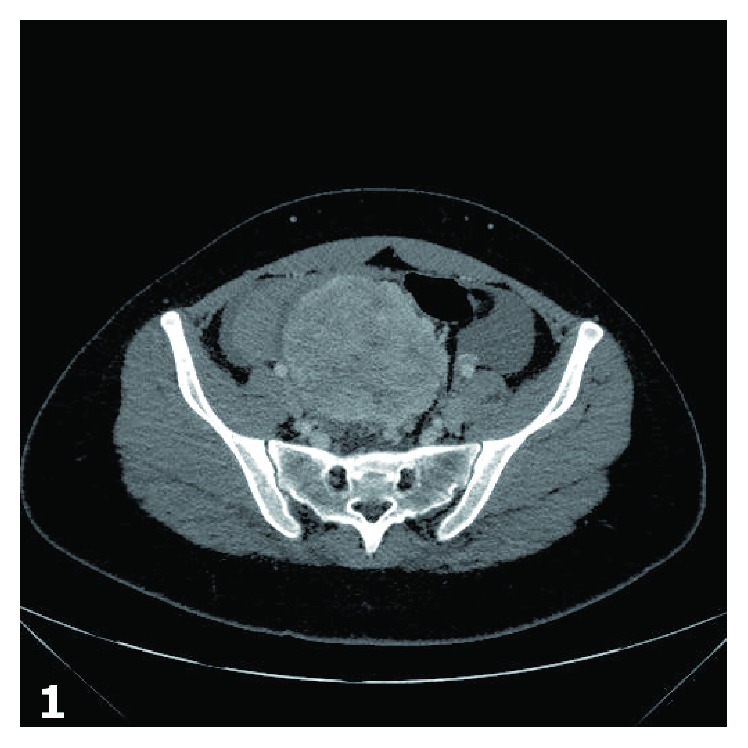
Computed tomography scan: There is a large irregular heterogeneous exophytic mass that appears to be arising from the right adnexa which intraoperatively arose from the serosa of the sigmoid colon.

A 21-year-old woman presented with acute onset of abdominal pain with recent hematochezia and constipation. The pain was initially described as being most severe in the suprapubic area, followed by diffuse involvement of the entire abdomen, and ultimately localizing to the right lower quadrant. The clinical differential diagnosis at this point included acute appendicitis, ovarian cyst with possible rupture, urinary tract infection, internal hemorrhoids, inflammatory bowel disease, and pancreatitis. An abdominal computed tomography (CT) scan demonstrated an irregular, right adnexal mass measuring 8.8 × 7.8 cm (Figure 1). A pelvic ultrasound also demonstrated a large right adnexal mass measuring 9.1 × 8.4 × 7.7 cm with internal vascularity. The mass was radiographically concerning for an adnexal tumor with possible ovarian torsion. The decision was made to perform a diagnostic laparoscopy and remove the adnexal mass. During the operation, the mass was noted to be an extrinsic sigmoid colon mass with active bleeding and hemoperitoneum (approximately 400 milliliters of blood) without involvement of the adnexa. The uterus, bilateral fallopian tubes, ovaries, and liver were grossly normal intraoperatively. The procedure was changed to an open laparotomy and the mass was resected along with the sigmoid colon and a low pelvic anastomosis was performed. The patient recuperated well after surgery. A CT scan was performed after six months to assess for residual or metastatic disease. The scan demonstrated multiple new ill-defined, low attenuating lesions in the liver, the largest measuring 6 cm in greatest dimension. A follow-up magnetic resonance imaging (MRI) scan showed several subtle, ill-defined areas of restricted diffusion that were not consistent with metastatic disease. Four years after surgery, the patient remains free of disease.

### 2.2. Macroscopic Findings

**Figure 2:**
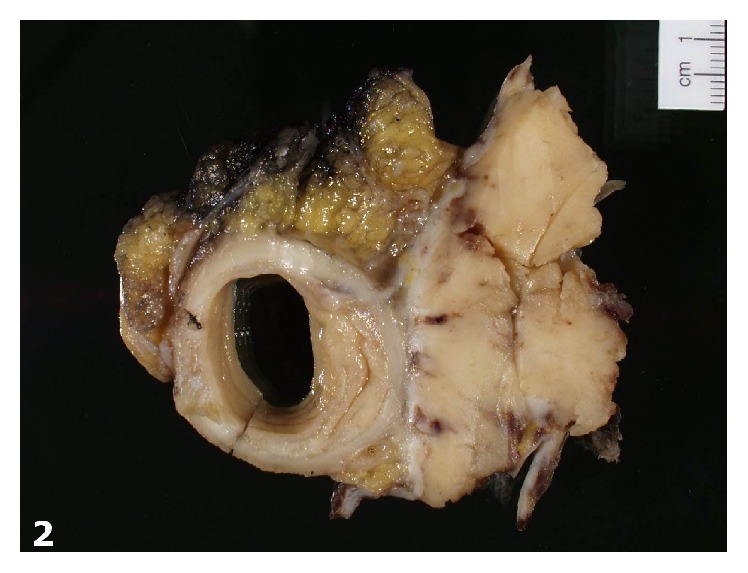
Gross photograph demonstrating a tan-white mass arising from the serosal surface of the sigmoid colon.

Grossly, the mass was fragmented and 16 × 11 × 9 cm in size (aggregate measurement). The mass was well-circumscribed but unencapsulated and appeared to arise from the serosal surface of the sigmoid colon (Figure 2). Cut surfaces were firm, tan-white to tan-yellow.

### 2.3. Microscopic Findings

**Figure 3:**
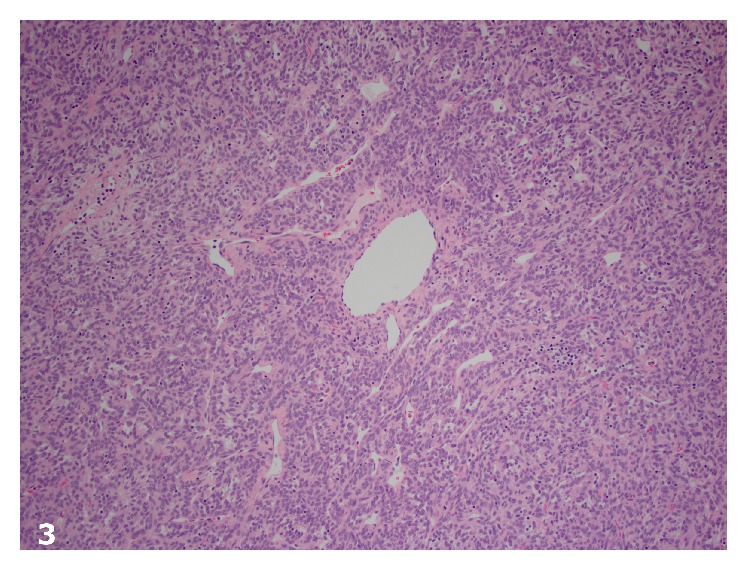
Hypercellular spindle cell neoplasm with hemangiopericytoma-like vasculature (H&E, original magnification ×100).

**Figure 4:**
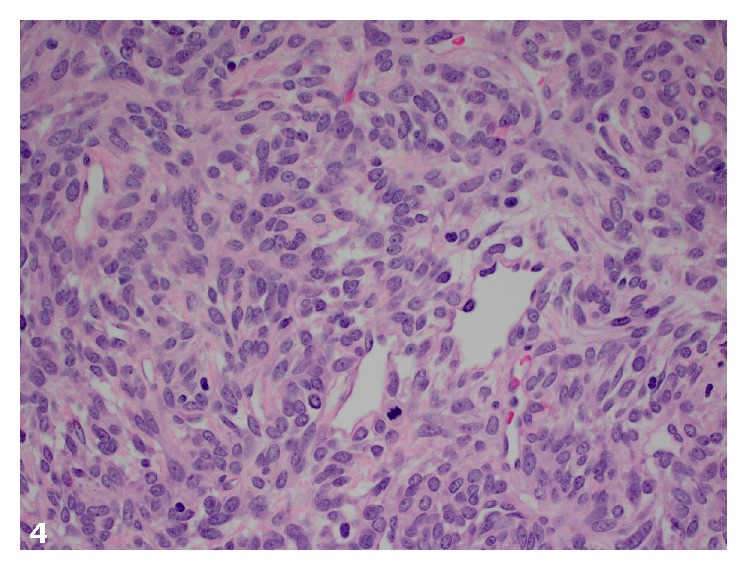
The tumor cells are ovoid to spindled with scant cytoplasm, indistinct borders, and vesicular nuclear chromatin. A mitotic figure is present in the lower-half of the field (H&E, original magnification ×400).

**Figure 5:**
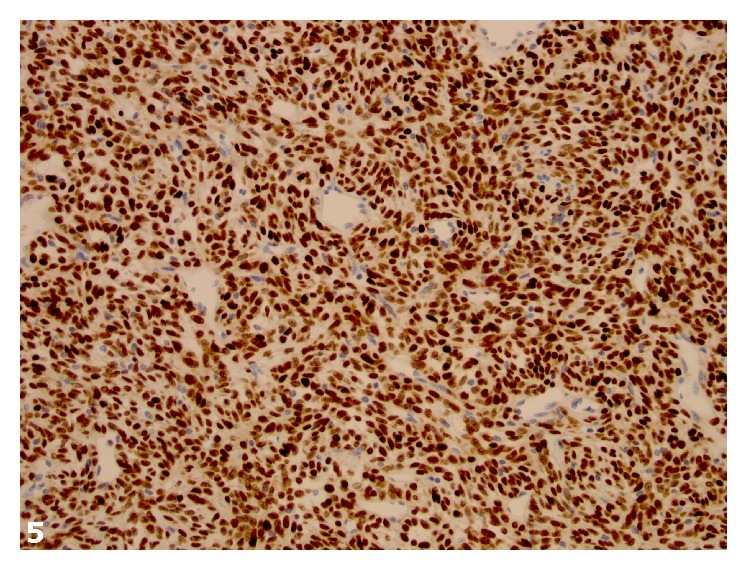
The neoplastic cells demonstrate strong nuclear positivity with antibody to STAT6 by immunohistochemistry (STAT6, original magnification ×200).

Histologically, the tumor was composed of spindle cells with predominantly hypercellular and focal hypocellular areas and prominent hemangiopericytoma-like vasculature. Cytologically, the spindled cells had a scant amount of cytoplasm, mild to moderate cytologic atypia and pleomorphism, and 8 mitotic figures per ten high-power fields (Figures 3 and 4). The tumor had a focally infiltrative edge and hemorrhage but no necrosis. Immunohistochemical stains showed strong and diffuse positivity for CD34 and patchy Bcl-2 positivity. The neoplastic cells were strongly and diffusely positive for STAT6 by immunohistochemistry (Figure 5). The tumor was negative for desmin, smooth muscle actin, inhibin, and CD117.

## 3. Discussion

Solitary fibrous tumor is a rare benign mesenchymal neoplasm originally described in the pleura, but now known to occur at nearly any extrapleural site [1–10]. Extrapleural solitary fibrous tumor most commonly occurs in adults with a wide age range of 20–70 years and tends to affect men and women equally [1–10]. Most tumors present as a slow growing painless mass or compress other anatomic structures in the vicinity of the mass [1].

Macroscopically, the majority of SFTs are well-circumscribed, partially encapsulated masses that are between 1 and 25 cm in size [1–7, 9, 10]. Cut surfaces are typically firm, tan-white, and multinodular and may have myxoid changes and hemorrhage [1, 5, 7, 10]. Tumor necrosis and infiltrative edges are usually seen in malignant examples [1, 7]. Histologically, SFTs demonstrate the so-called “patternless pattern” which is characterized by alternating hypercellular and hypocellular zones with interspersed collagen which may be hyalinized and keloid-like. Perivascular hyalinization may be seen. Cytologically, the cells are usually ovoid to spindled with limited cytoplasm, indistinct borders, and vesicular nuclear chromatin. Myxoid areas, areas of fibrosis, and mast cells are common features. Criteria for malignancy have been established and include ≥4 mitotic figures per 10 high-power fields, increased cellularity, variable cytologic atypia, tumor necrosis, and/or infiltrative margins. The most prognostic feature seems to be the mitotic rate [1–7, 9–12]. Some cases demonstrate abrupt transition to a high-grade sarcoma which has been termed “dedifferentiation” similar to that seen in other soft tissue tumors (i.e., liposarcoma) and act as aggressive sarcomas [11]. Immunophenotypically, a vast majority (90–95%) of these tumors are CD34 positive and most also express Bcl-2. They may be positive for epithelial membrane antigen (EMA) and smooth muscle actin (SMA) in approximately 20–35% of cases which may be a diagnostic pitfall because synovial sarcoma and smooth muscle tumors are common in the morphologic differential diagnosis [1–7, 9, 10]. Recently, it has been shown that diminished expression of CD34 and increased expression of IGF2 are significantly associated with malignant transformation and may be useful in the future as a therapeutic target [12].

The histologic differential diagnosis for extrapleural solitary fibrous tumor is broad [4]. Leiomyomas and leiomyosarcomas have more abundant eosinophilic cytoplasm and larger nuclei, and a majority will express at least one muscle marker (usually smooth muscle actin or desmin) [4, 7]. Neurofibroma may enter the differential diagnosis and can be excluded by the absence of staining with S-100 protein [4]. In the soft tissues and dermis, dermatofibrosarcoma protuberans (DFSP) may be CD34 positive but lacks the characteristic vascular pattern of SFT. Additionally, a recurring translocation t(17;22)(q22;q13) involving the collagen type-1 *α*1 gene on chromosome 17 and platelet-derived growth factor *β* gene on chromosome 22 is seen in DFSP but not SFT [5, 7]. Some other site-specific tumors in the differential diagnosis include fibromatosis, gastrointestinal stromal tumor and desmoplastic mesothelioma in retroperitoneal and pelvic cases [5, 7]. Meningioma and mesoblastic nephroma may be considered in meningeal and renal cases, respectively [5]. The correct diagnosis of SFT can usually be made by morphology and the prudent use of immunohistochemistry [5].

A recurring and nonrandom fusion of two genes has been recently identified in solitary fibrous tumor and involves the NGFI-A-binding protein 2 (NAB2) and STAT6 genes, both located at chromosomal region 12q13. This fusion event is thought to represent an initial tumorigenic event in SFT [13–15]. There are variant fusions that differ with regard to clinicopathologic features. The most common fusion variant, NAB2ex4-STAT6ex2/3, is found in the classic pleuropulmonary SFT with diffuse fibrosis, has mostly benign behavior, and is found in older individuals (median age: 69 years). The second most common fusion variant, NAB2ex6-STAT6ex16/17, is found in “typical” SFTs from the soft tissue; the tumors tend to be more aggressive, and they occur in younger individuals (median age: 47 years) [16]. The discovery of this fusion has been utilized immunohistochemically as well, and STAT6 is an excellent and specific marker for SFTs, being positive in approximately 98% [17].

The prognosis for extrathoracic solitary fibrous tumor is difficult to assess and can be unpredictable but most cases behave in a benign fashion [1–3, 5–9]. Approximately 10–36% of these tumors behave aggressively and local and/or distant recurrences may occur years after the primary diagnosis [1, 3, 5, 7, 9]. At this time, there is no definitive correlation between histology and behavior but malignant features histologically (in particular, mitotic counts) continue to be the best predictor of poor outcome [1, 5, 7–9]. Site of the tumor is also an indicator of behavior as those tumors in the limbs tend to behave in a benign fashion whereas those located in the mediastinum, abdomen, pelvis, retroperitoneum, and/or meninges behave more aggressively [1, 3, 8, 9]. Tumor size over 10 cm and positive margins are also predictive of a more aggressive tumor [1]. When metastases do occur, they most commonly occur in the lungs, liver, and bones [1, 7]. Close clinical follow-up is prudent in all extrapleural SFTs [5, 7].

Herein, we have reported a case of a malignant solitary fibrous tumor, by current histologic criteria, in a rare extrapleural location: the serosal surface of the sigmoid colon. This is a relatively uncommon site with only two other reported cases, to our knowledge, in the sigmoid mesocolon [7, 18].
